# An External-Validated Algorithm to Predict Postoperative Pneumonia Among Elderly Patients With Lung Cancer After Video-Assisted Thoracoscopic Surgery

**DOI:** 10.3389/fonc.2021.777564

**Published:** 2021-12-14

**Authors:** Yanping Song, Jingjing Liu, Mingxing Lei, Yanfeng Wang, Qiang Fu, Bailin Wang, Yongxin Guo, Weidong Mi, Li Tong

**Affiliations:** ^1^ Anesthesia and Operation Center, The First Medical Center of Chinese People’s Liberation Army (PLA) General Hospital, Beijing, China; ^2^ Department of Anesthesia, 922 Hospital of People’s Liberation Army (PLA), Hengyang, China; ^3^ Department of Anesthesia, Beijing Corps Hospital of Chinese People’s Armed Police Force, Beijing, China; ^4^ The National Clinical Research Center for Orthopedics, Sports Medicine & Rehabilitation, The First Medical Center of Chinese People’s Liberation Army (PLA) General Hospital, Beijing, China; ^5^ Department of Orthopedic Surgery, Hainan Hospital of Chinese People’s Liberation Army (PLA) General Hospital, Sanya, China; ^6^ Chinese People’s Liberation Army (PLA) Medical School, Beijing, China; ^7^ Department of Anesthesia, Xiangya Hospital, Central South University, Changsha, China; ^8^ Department of Thoracic Surgery, Hainan Hospital of Chinese People’s Liberation Army (PLA) General Hospital, Sanya, China

**Keywords:** lung cancer, video-assisted thoracoscopic surgery, postoperative pneumonia, prediction model, risk factors

## Abstract

The aim of the study was to develop an algorithm to predict postoperative pneumonia among elderly patients with lung cancer after video-assisted thoracoscopic surgery. We analyzed 3,009 patients from the Thoracic Perioperative Database for Geriatrics in our hospital and finally enrolled 1,585 elderly patients (age≧65 years) with lung cancer treated with video-assisted thoracoscopic surgery. The included patients were randomly divided into a training group (*n* = 793) and a validation group (*n* = 792). Patients in the training group were used to develop the algorithm after screening up to 30 potential risk factors, and patients in the validation group were used to internally validate the algorithm. External validation of the algorithm was achieved in the external validation dataset after enrolling 165 elderly patients with lung cancer treated with video-assisted thoracoscopic surgery from two hospitals in China. Of all included patients, 9.15% (145/1,585) of patients suffered from postoperative pneumonia in the Thoracic Perioperative Database for Geriatrics, and 10.30% (17/165) of patients had postoperative pneumonia in the external validation dataset. The algorithm consisted of seven variables, including sex, smoking, history of chronic obstructive pulmonary disease (COPD), surgery duration, leukocyte count, intraoperative injection of colloid, and intraoperative injection of hormone. The C-index from the receiver operating characteristic curve (AUROC) was 0.70 in the training group, 0.67 in the internal validation group, and 0.71 in the external validation dataset, and the corresponding calibration slopes were 0.88 (95% confident interval [CI]: 0.37–1.39), 0.90 (95% CI: 0.46–1.34), and 1.03 (95% CI: 0.24–1.83), respectively. The actual probabilities of postoperative pneumonia were 5.14% (53/1031) in the low-risk group, 15.07% (71/471) in the medium-risk group, and 25.30% (21/83) in the high-risk group (*p* < 0.001). The algorithm can be a useful prognostic tool to predict the risk of developing postoperative pneumonia among elderly patients with lung cancer after video-assisted thoracoscopic surgery.

## Introduction

Lung cancer is one of the severe worldwide health problems. The number of lung cancer patients is globally growing and lung cancer is responsible for the most common cause of cancer deaths (1). More explicitly, lung cancer is the most commonly diagnosed cancer, which accounts for not only 11.6% of the total cancer cases but also 18.4% of the total cancer deaths when male and female were combined together according to the Global Cancer (GLOBOCAN) report in 2018 (2). In this year, the GLOBOCAN reported that lung cancer had 2.09 million new cases and 1.76 million deaths among 185 countries.

Video-assisted thoracoscopic surgery is an effective and safe intervention to treat the majority of patients with early-stage lung cancer since a multitude of clinical evidence has demonstrated that the surgery was capable of realizing shorter length of hospitalization (3), less intraoperative blood loss, less chest tube drainage (4), less trauma, less postoperative pain (5), better postoperative recovery (6), better quality of life (5), and comparable survival outcome (7) or even better survival outcome (8) as compared with open surgery for lung cancer patients (9, 10). Besides, video-assisted thoracoscopic surgery was similar to robot-assisted thoracoscopic surgery in terms of medium-term survival prognosis (11). Thus, video-assisted thoracoscopic surgery is considered as the gold standard for lung cancer surgery and will remain so for the foreseeable future (9).

Unfortunately, the postoperative complication of video-assisted thoracoscopic surgery is still a major challenge. A multicenter prospective clinical trial has reported that the rate of postoperative complication could be up to 28.6% after video-assisted thoracoscopic surgery (12). A prospective observational study reported that the rate of postoperative pulmonary complication could be 7.40% (13) and it is related to longer hospitalization, higher frequency of intensive therapy unit admission, and higher hospital mortality (14). Moreover, the incidence of postoperative pneumonia could be as high as 19.7% after video-assisted thoracoscopic surgery among lung cancer patients (15). Thus, identification and modification of risk factors associated with postoperative pneumonia could be very beneficial to predict and subsequently prevent the complication in advance. However, studies about potential risk factors for postoperative pneumonia were very limited, especially among elderly patients. A prospective observational study found that current smoking might be an independent risk factor for postoperative pulmonary complications after analyzing a relative small series of lung cancer patients (*n* = 285) undergoing video-assisted thoracoscopic surgery (14). Therefore, it is really warranted to further investigate potential contributors relating to postoperative pneumonia among lung cancer patients after video-assisted thoracoscopic surgery. Although there has been a model to predict the survival outcome among lung cancer patients after surgery (16), prediction models to predict the postoperative complication were scarce. Thus, the development of a model to predict the probability of postoperative pneumonia in advance would be of great clinical significance.

Therefore, we aimed to develop an algorithm to predict postoperative pneumonia especially among elderly patients with lung cancer after video-assisted thoracoscopic surgery, and further investigate major contributors to postoperative pneumonia so that effective preventive strategies could be conducted earlier.

## Patients and Methods

### Patient’s Selection and Study Design

**Figure 1:**
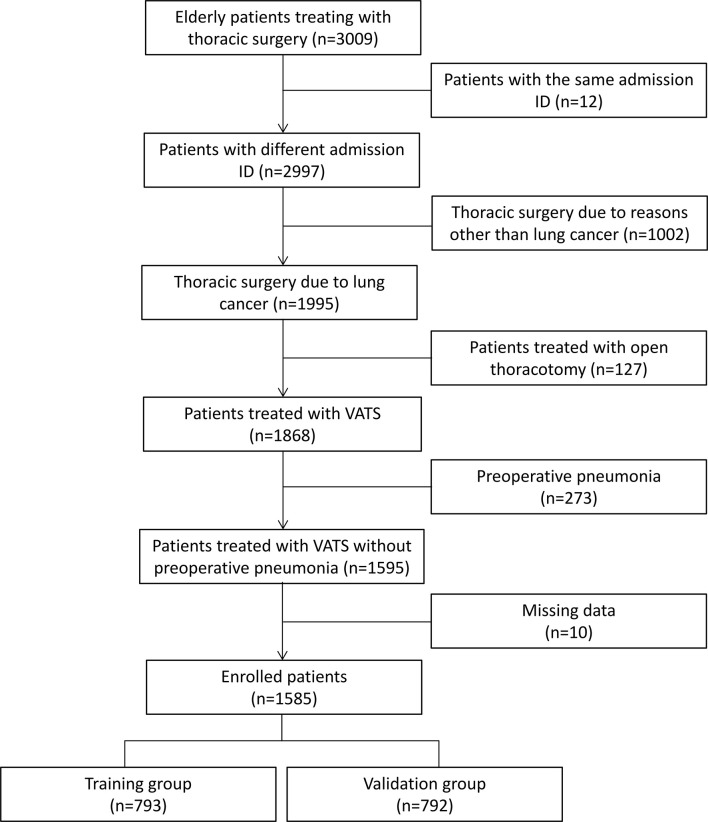
Patient’s flow chart. The enrolled patients were randomly divided into the training group and the validation group. VATS indicates video-assisted thoracoscopic surgery.

We retrospectively analyzed 3,009 patients treated with thoracic surgery from the Thoracic Perioperative Database for Geriatrics in the First Medical Center of Chinese PLA General Hospital (Beijing), and we finally enrolled 1,585 elderly patients with lung cancer treated with video-assisted thoracoscopic surgery between January 2012 and December 2019. The Thoracic Perioperative Database for Geriatrics was established at the First Medical Center of Chinese PLA General Hospital using the WebService interface. It automatically extracted data from the electronic medical record system, clinical laboratory system, medical image management system, radiation information management system, blood transfusion management system, and nurse workstation. Patient’s diagnoses, examinations, prescriptions, laboratory tests, medical insurances, and operation information were extracted. Then, all data (100%) were rechecked and reconfirmed by two medical workers at least and contradictory data were reviewed and confirmed after discussions. Data standardization complied with the Clinical Data Interchange Standards Consortium (CDISC). In the present study, patients were included if they were elderly patients (age≧65 years) with tissue-proved diagnosis of lung cancer and treated with video-assisted thoracoscopic surgery. Patients were excluded in the analysis, if (1) patients had the same admission ID, (2) received thoracic surgery due to esophageal cancer, gastric antral cancer, bullae of lung, and other reasons, (3) treated with open thoracotomy, (4) preoperative pneumonia confirmed by computed tomography (CT), and (5) missing data. **Figure 1** shows the patient’s flow chart.

The enrolled patients were randomly classified into two groups: namely, the training group (*n* = 793) and the validation group (*n* = 792). The randomization of patients was computer-based and achieved with the ratio of 1:1, which was consistent with other studies (17–19). Patients in the training group were conducted to develop the algorithm and patients in the validation group were used to internally validate the predictive performance of the algorithm. External validation of the algorithm was achieved in the external validation dataset after enrolling 165 patients from the Hainan Hospital of Chinese PLA General Hospital (Sanya, Hainan) and Xiangya Hospital of the Central South University (Changsha, Hunan) between January 2019 and September 2021 according to the inclusive and exclusive criteria.

The study was conducted in accordance with the Declaration of Helsinki. The study was approved by the Ethics Committee Board of the First Medical Center of Chinese PLA General Hospital (No. S2019-311-03) and patient’s written consents were waived because all data were anonymized and the study was retrospective in nature.

### Surgical Indication and Procedures

The main surgical indications were as follows: (1) lung cancer at stage I, II, and III; (2) no cancer involvement in the chest wall and mediastinum; (3) no recent myocardial infarction and tendency of severe bleeding; and (4) tolerable to single lung ventilation. Generally, patients received general anesthesia and then were placed in lateral position. Operation was conducted with video-assisted thoracoscopic surgery, which was performed with one access window and one operation port. After surgery, a closed drainage was placed at the eighth intercostal space and a vacuum sealing drainage was placed at the operation port.

### Analysis of Patient’s Characteristics

Based on the literature and available variables in the dataset, we collected 30 potential characteristics and the 30 variables could be divided into (1) basic characteristics including age, sex (male *vs.* female), body mass index (BMI, kg/m^2^), smoking (no *vs.* yes), and alcohol drinker (no *vs.* yes); (2) comorbidities including hypertension (yes *vs.* no), diabetes (yes *vs.* no), coronary heart disease (yes *vs.* no), myocardial infarction (yes *vs.* no), cerebrovascular disease (yes *vs.* no), asthma (yes *vs.* no), chronic obstructive pulmonary disease (COPD) (yes *vs.* no), renal function insufficiency (yes *vs.* no), and peptic ulcer (yes *vs.* no); (3) laboratory testing including serum albumin (g/L), leukocyte count (×10^9^/L), red blood cell volume distribution width (RDW, %), serum glucose (mmol/L), blood urea nitrogen (mmol/L), blood potassium (mEq/L), blood sodium (mEq/L), serum creatinine (μmol/L), and total bilirubin (μmol/L); (4) preoperative medication history including angiotensin-converting enzyme inhibitor (ACEI) drug (yes *vs.* no), statin drug (yes *vs.* no), and calcium channel blocker (yes *vs.* no); and (5) variables immediately before or during surgery including preoperative mean artery pressure (MAP) (mmHg), surgery duration (minutes, min), intraoperative administration of colloid (ml) (0 *vs.* >0 and ≦500 *vs.* >500), and intraoperative administration of hormone (yes *vs.* no). All laboratory testing data were collected before surgery. MAP was evaluated just before surgery. Surgery duration was defined as the time interval between skin incision and suture. Hormone injection included dexamethasone and methylprednisolone. Besides, hospitalization expenses of all patients were also recorded in the study. Hospitalization expense indicated all costs that were spent to treat the disease during the hospitalization. Hospitalization expense was converted from renminbi to dollar according to the current exchange rate. We compared the patient’s basic and clinical demographics according to the presence of postoperative pneumonia.

### The Main Prognostic Parameter: Postoperative Pneumonia

Postoperative pneumonia refers to abnormal radiographic findings including new infiltrations being found in postoperative chest x-ray or CT and patients had the following one or more symptoms postoperatively during hospitalization (20): (1) patients had new or progressive respiratory symptoms, such as coughing and expectoration; (2) patients had a vital sign of fever >38°C or hypothermia; (3) patients had a physical examination of lung consolidation signs or moist rale; (4) patients had a white cell count of more than 10 × 10^9^/L or less than 4 × 10^9^/L or a new rise in the C-reactive; and (5) patients had pathogen isolation derived from blood culture or sputum.

### Algorithm Development

In the training group, we used the Least Absolute Shrinkage and Selection Operator (LASSO) logistic regression model to screen the above-mentioned 30 variables. The LASSO method was a penalized regression tool with the ability of selecting variables *via* minimizing the comparatively irrelevant variables’ coefficient to 0 and subsequently excluding unwarranted variables. The 10-fold cross-validation was conducted to adjust the parameter λ to further improve the accuracy of the LASSO regression model. The LASSO-identified variables were included to develop the algorithm. The estimates of the algorithm and corresponding intercept were obtained from the maximum likelihood estimates according to the multiple logistic regression analysis and then the algorithm was created based on the logistic regression equation: P(Y=1) = *e^intercept^* ^+^ *^ax1^* ^+^ *^bx2^* ^+^ *^cx3^* ^…+^ *^ixn^*/(1 + *e^intercept^* ^+^ *^ax1^* ^+^ *^bx2^* ^+^ *^cx3^* ^…+^ *^ixn^*). In the algorithm, *a* to *i* were the maximum likelihood estimates and *x1* to *xn* were the LASSO-identified significant variables. P(Y=1) refers to the predicted probability of developing postoperative pneumonia among elderly patients with lung cancer treated with video-assisted thoracoscopic surgery.

### Algorithm Validation

The internal validation of the predictive performance of the algorithm was conducted in the validation group and the training group, and the external validation was performed in the external validation dataset. The predictive ability of the algorithm was evaluated using the discrimination and calibration abilities.

The discrimination ability was the algorithm’s capability to identify patients with postoperative pneumonia and patients without it. The discrimination ability was measured by the AUROC and the discrimination slope. The discrimination slope was the difference between the mean predicted probabilities of developing postoperative pneumonia in patients with (positive) and without (negative) actual postoperative pneumonia. Besides, the algorithm’s correct classification rate, sensitivity, specificity, false-positive rate, and false-negative rate were calculated and presented in the study.

The calibration ability was the algorithm’s capability to confirm the consistency between the observed and the predicted probability of developing postoperative pneumonia. The calibration ability was measured by the calibration slope and the *R* ^2^ which was calculated by the goodness-of-fit test. Calibration curve refers to the decile plot of the observed probability of developing postoperative pneumonia against the predicted probability of postoperative pneumonia in each decile. If the slope of the calibration curve is close to 1 and intercept is close to 0, it indicates good calibration ability. The *R^2^* of the fitted line in the calibration curve was also presented in the study based on the goodness-of-fit test.

### Statistical Analysis

We presented statistical descriptions of all patients. If the continuous data were normally distributed, they were shown as mean ± standard deviation (SD); if the continuous data were not normally distributed, they were shown as median and interquartile range (IQR). The categorical data were presented in proportion. The Wilcoxon test, Chi-square test, and continuity adjusted Chi-square test were used to compare differences of characteristics between patients with and without postoperative pneumonia. The Pearson correlation analysis was used to analyze the correlation between the seven variables and the main prognostic parameter (postoperative pneumonia), and the correlation matrix of the Pearson correlation coefficients was visualized by the heat map. Three different risk groups were achieved according to the cutoff of the algorithm and the Chi-square test was used to compare the difference between the actual probabilities of developing postoperative pneumonia among the three risk groups. Principal components analysis was performed to explore potential major contributors to postoperative pneumonia and principal equations were given according to eigenvectors. All analyses were performed using SAS 9.4 software (SAS Institute Inc., Cary, NC) and the heat map was visualized using R version 3.5.3 for Windows XP.

## Results

### Patient’s Clinical Demographics

Of all the included patients in the Thoracic Perioperative Database for Geriatrics, 9.15% (145/1,585) of patients developed postoperative pneumonia. Subgroup analysis of patients was performed based on the presence of postoperative pneumonia (**Table 1**). In the study, patients with postoperative pneumonia were more likely to be male (68.97% *vs.* 56.18%, *p* = 0.03) and smoker (46.21% *vs.* 37.78%, *p* = 0.047) as compared to patients without it. Besides, patients with postoperative pneumonia had a higher level of preoperative leukocyte count (×10^9^/L) (6.36 [5.20, 7.40] *vs.* 5.92 [4.97, 6.97], *p* = 0.009) and serum creatinine (μmol/L) (75.30 [63.60, 84.40] *vs.* 71.70 [61.30, 81.30], *p* = 0.041), a higher rate of preoperative calcium channel blocker usage (35.17% *vs.* 25.49%, *p* = 0.012), and a longer surgery duration (min) (175.00 [145.00, 223.00] *vs.* 150.00 [113.00, 187.00], *p* < 0.001) than those without it. Regarding intraoperative drug administration, hormones were administered more among the patients with postoperative pneumonia than that among the patients without postoperative pneumonia (86.21% *vs.* 74.44%, *p* = 0.002) and so was colloid (*p* < 0.001). Patients with postoperative pneumonia had a significantly higher hospitalization expense as compared with those without postoperative pneumonia ($) (11,537.93 [9,488.58, 14,088.16] *vs.* 10,069.85 [8,297.86, 11,968.01], *p* < 0.001).

**Table 1:** Patient’s basic and clinical demographics between patients with and without postoperative pneumonia.

Characteristics	Postoperative pneumonia		*p*
	Yes (*n* = 145)	No (*n* = 1440)	
Age (years, median [IQR])	68.00 [66.00, 71.00]	69.00 [66.00, 72.00]	0.085^a^
Sex			
Male	68.97% (100/145)	56.18% (809/1440)	0.003^b^
Female	31.03% (45/145)	43.82% (631/1440)	
BMI (kg/m^2^, median [IQR])	24.99 [23.14, 26.37]	24.43 [22.48, 26.53]	0.236^a^
Smoking			
No	53.79% (78/145)	62.22% (896/1440)	0.047^b^
Yes	46.21% (67/145)	37.78% (544/1440)	
Alcohol drinker			
No	68.28% (99/145)	72.08% (1038/1440)	0.332^b^
Yes	31.72% (46/145)	27.92% (402/1440)	
Comorbidities			
Hypertension (%)	45.52% (66/145)	42.71% (615/1440)	0.515^b^
Diabetes (%)	18.62% (27/145)	23.13% (333/1440)	0.217^b^
Coronary heart disease (%)	12.41% (18/145)	12.36% (178/1440)	0.985^b^
Myocardial infarction (%)	2.76% (4/145)	0.83% (12/1440)	0.076^c^
Cerebrovascular disease (%)	14.48% (21/145)	11.11% (160/1440)	0.224^b^
Asthma (%)	1.38% (2/145)	0.63% (9/1440)	0.604^c^
COPD	3.45% (5/145)	2.22% (32/1440)	0.520^c^
Renal function insufficiency (%)	0.69% (1/145)	0.83% (12/1440)	1.000^c^
Peptic ulcer (%)	0.69% (1/145)	0.83% (12/1440)	1.000^c^
Laboratory testing			
Serum albumin (g/L, mean ± SD)	41.50 [39.20, 43.80]	41.50 [39.50, 43.60]	0.568^a^
Leukocyte count (×10^9^/L, median [IQR])	6.36 [5.20, 7.40]	5.92 [4.97, 6.97]	0.009^a^
RDW (%, median [IQR])	12.70 [12.30, 13.20]	12.70 [12.30, 13.20]	0.511^a^
Serum glucose (mmol/L, median [IQR])	5.03 [4.63, 5.73]	5.00 [4.62, 5.61]	0.592^a^
Blood urea nitrogen (mmol/L, median [IQR])	5.09 [4.39, 6.06]	5.16 [4.33, 6.10]	0.970^a^
Blood potassium (mEq/L, mean ± SD)	4.08 [3.88, 4.28]	4.10 [3.88, 4.32]	0.604^a^
Blood sodium (mEq/L, median [IQR])	142.70 [141.20, 144.30]	142.70 [141.20, 144.0]	0.726^a^
Serum creatinine (μmol/L, median [IQR])	75.30 [63.60, 84.40]	71.70 [61.30, 81.30]	0.041^a^
Total bilirubin (μmol/L, median [IQR])	11.10 [8.30, 14.10]	10.90 [8.50, 13.80]	0.842^a^
Preoperative medication			
ACEI drug (%)	2.07% (3/145)	2.50% (36/1440)	0.970^c^
Statin drug (%)	8.97% (13/145)	5.90% (85/1440)	0.144^b^
Calcium channel blocker (%)	35.17% (51/145)	25.49% (367/1440)	0.012^b^
Preoperative MAP (mmHg, median [IQR])	95.33 [88.00, 103.00]	96.00 [88.67, 103.33]	0.607^a^
Surgery duration (min, median [IQR])	175.00 [145.00, 223.00]	150.00 [113.00, 187.00]	<0.001^a^
Intraoperative drug administration			
Colloid (ml)			
0	28.28% (41/145)	39.17% (564/1440)	<0.001^b^
>0 and ≦500	59.31% (86/145)	55.56% (800/1440)	
>500	12.41% (18/145)	5.28% (76/1440)	
Hormone (%)^d^	86.21% (125/145)	74.44% (1072/1440)	0.002^b^
Hospitalization expense ($, median [IQR])	11,537.93 [9,488.58, 14,088.16]	10,069.85 [8,297.86, 11,968.01]	<0.001^a^

a The Wilcoxon test.

b the Chi-square test.

c the continuity adjusted Chi-square test.

d the hormones included methylprednisolone and/or dexamethasone.

IQR, Interquartile range; BMI, Body mass index; COPD, Chronic obstructive pulmonary disease; SD, Standard deviation; min, minutes; RDW, Red blood cell volume distribution width; ACEI, Angiotensin-converting enzyme inhibitor; MAP, Mean artery pressure.

### Algorithm Development

**Figure 2:**
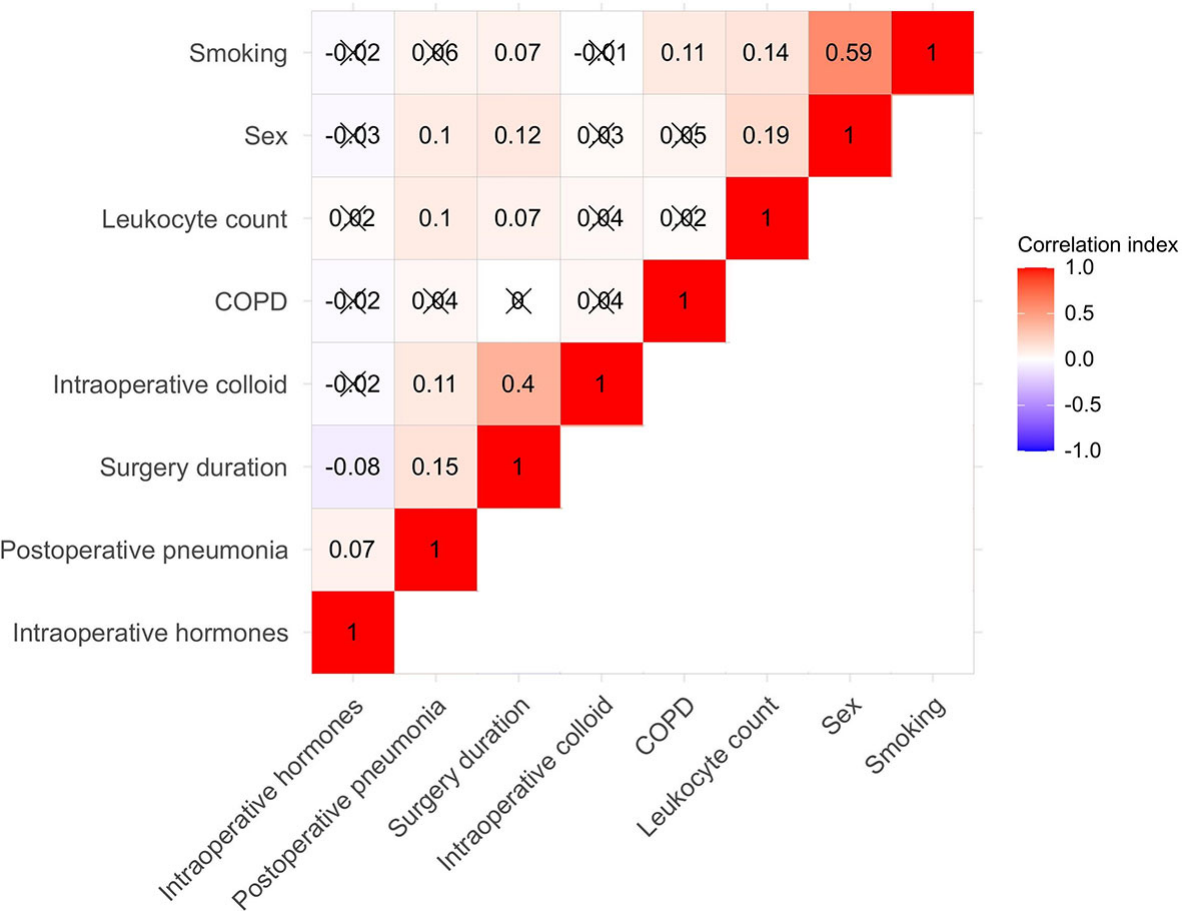
Heat map for the seven algorithm-included variables and postoperative pneumonia. Correlation matrix was shown and the Pearson correlation was given in each small square. Red represents positive correlation, white represents no correlation, and blue represents negative correlation. COPD indicates chronic obstructive pulmonary disease.

In the training group, the LASSO regression model (10-fold cross-validation) selected five variables, including sex, surgery duration, leukocyte count, intraoperative administration of colloid, and intraoperative administration of hormone, all of which were included to develop the algorithm. Besides, according to previous studies, we also added smoking and history of COPD into the algorithm. The Pearson correlation analysis further confirmed the relationship between the seven variables and the outcome variable, and the correlation matrix of the Pearson correlation coefficients was presented by heat map (**Figure 2**). It further demonstrated that the five LASSO-selected variables were significantly and positively related to postoperative pneumonia, which meant that male sex, longer surgery duration, higher leukocyte count, intraoperative use of colloid, and intraoperative use of hormone were associated with a higher risk of developing postoperative pneumonia.

According to the estimates of each enrolled variable from the multiple logistic regression, the equation was shown as follows (**Table 2**). The predicted probability of postoperative pneumonia was P (Y=1) = e^x^/(1+ e^x^), _X_= -6.03 + 0.60*sex + 0.01*smoking + 0.57*history of COPD + 0.006*surgery duration + 0.16*leukocyte count + 0.31*intraoperative administration of colloid + 0.86*intraoperative administration of hormone. A calculator was created to promote the application of the algorithm (**Supplementary File 1**). An example about how to use the algorithm was given as follows: If a male (1 point) elderly patient with lung cancer is treated with video-assisted thoracoscopic surgery, if he was a smoker (1 point) with a medical history of COPD (1 point), if the surgical duration was 120 min and his preoperative leukocyte count was 9.09×10^9^/L, and if he received an intraoperative injection of colloid (500 ml, 2 points) and hormone (1 point), his predicted probability of developing postoperative pneumonia was P (Y=1) = *e^x^*/(1+*e* ^x^) =*e^x^*/(1+ *e^x^*) =23.23% (*X*=-6.03 + 0.60 * 1 + 0.01 * 1 + 0.57 * 1 + 0.006 * 120 + 0.16 * 9.09 + 0.31 * 2 + 0.86 * 1=-1.1956).

**Table 2:** An algorithm to predict postoperative pneumonia among elderly patients with lung cancer after video-assisted thoracoscopic surgery.

Included variables	Estimates^a^
Intercept	-6.03
Sex (male indicates 1 point; female indicates 0 point)	0.60
Smoking (yes indicates 1 point; no indicates 0 point)	0.01
History of COPD (yes indicates 1 point; no indicates 0 point)	0.57
Surgery duration (min)	0.006
Leukocyte count (×10^9^/L)	0.16
Intraoperative administration of colloid (0 indicates 1 point; >0 and ≦500 indicates 2 points; >500 indicates 3 points)	0.31
Intraoperative administration of hormone (yes indicates 1 point; no indicates 0 point)	0.86

The algorithm was created as follows:

P (Y = 1) = e^x^/(1+e^x^), x=-6.03 + 0.60 * sex + 0.01 * smoking + 0.57 * COPD + 0.006 * sugery duration +0.16 * leukocyte count + 0.31 * intraoperative colloid + 0.86 * intraoperative hormone. P(Y = 1) indicates the predicted probability of developing postoperative pneumonia.

a Maximum likelihood estimates from the logistic regression analysis.

min, minutes; COPD, chronic obstructive pulmonary disease.

### Algorithm Validation

**Figure 3:**
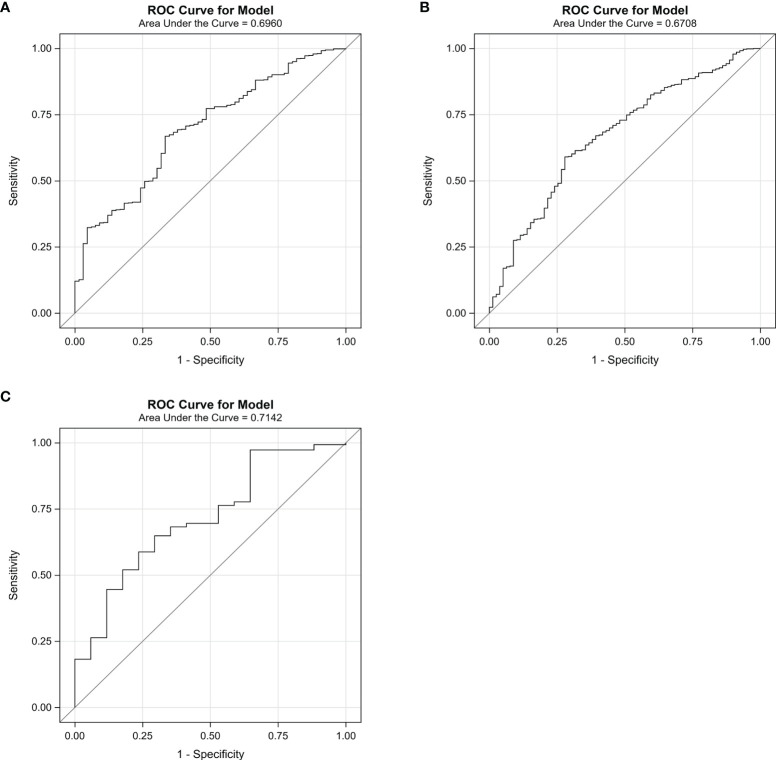
The area under the receiver operating characteristic curve (AUROC) for the algorithm in the training group **(A)**, the internal validation group **(B)**, and the external validation dataset **(C)**. The curve was plotted with sensitivity against 1 − specificity. The C-index was 0.70 in the training group, 0.67 in the internal validation group, and 0.71 in the external validation group.

**Figure 4:**
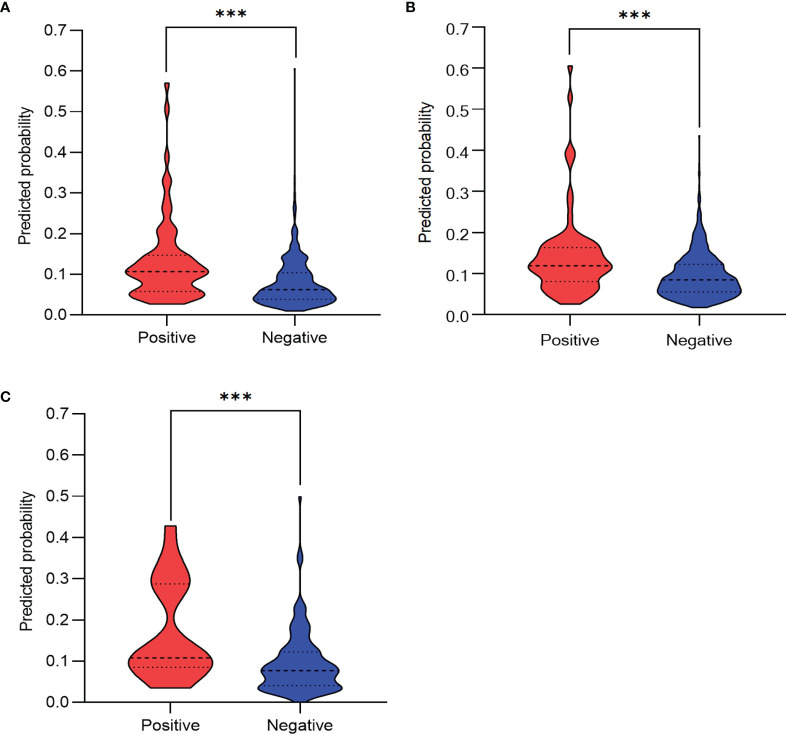
Discrimination slope for the algorithm in the training group **(A)**, the internal validation group **(B)**, and the external validation dataset **(C)**. The violin plots were drawn with predicted probability of developing postoperative pneumonia against actual status (positive indicates patients with postoperative pneumonia; negative indicates patients without postoperative pneumonia). The discrimination slope was 0.06 (95%CI: 0.04-0.07, p < 0.001 ,Wilcoxon test) in the training group, 0.05 (95% CI: 0.03–0.06, *p* < 0.001, Wilcoxon test) in the internal validation group, and 0.08 (95% CI: 0.04–0.12, *p* < 0.001, Wilcoxon test) in the external validation dataset. ***P < 0.001.

The predictive performance of the algorithm was validated using the discrimination and calibration ability in the training, internal validation, and external validation dataset. In the external validation dataset, 10.30% (17/165) of patients had postoperative pneumonia. The C-index from the AUROC was 0.70 in the training group (**Figure 3A**), 0.67 in the internal validation group (**Figure 3B**), and 0.71 in the external validation dataset (**Figure 3C**). The discrimination slopes were 0.06 (95% CI: 0.04-0.07) in the training group (**Figure 4A**), 0.05 (95% CI: 0.03-0.06) in the internal validation group (**Figure 4B**), and 0.08 (95% CI: 0.04-0.12) in the external validation dataset (**Figure 4C**). The corresponding correct classification rates were 64.10%, 61.50%, and 78.80%, respectively. More details are presented in **Table 3**, which also showed that the algorithm had acceptable sensitivity and specificity and low false-negative rate.

**Table 3:** Evaluation of the discrimination ability of the algorithm.

Groups	AUROC	Slope	95% CI	CCR	Sensitivity	Specificity	False POS	False NEG
Training group	0.70	0.06	0.04–0.07	64.10%	65.20%	64.00%	85.90%	4.70%
Internal validation	0.67	0.05	0.03–0.06	61.50%	63.30%	61.30%	84.70%	6.20%
External validation	0.71	0.08	0.04–0.12	78.80%	41.20%	83.10%	78.10%	7.50%

AUROC, the area under the receiver operating characteristic curve; CI, confident interval; CCR, correct classification rate; POS indicates positive; NEG indicates negative.

**Figure 5:**
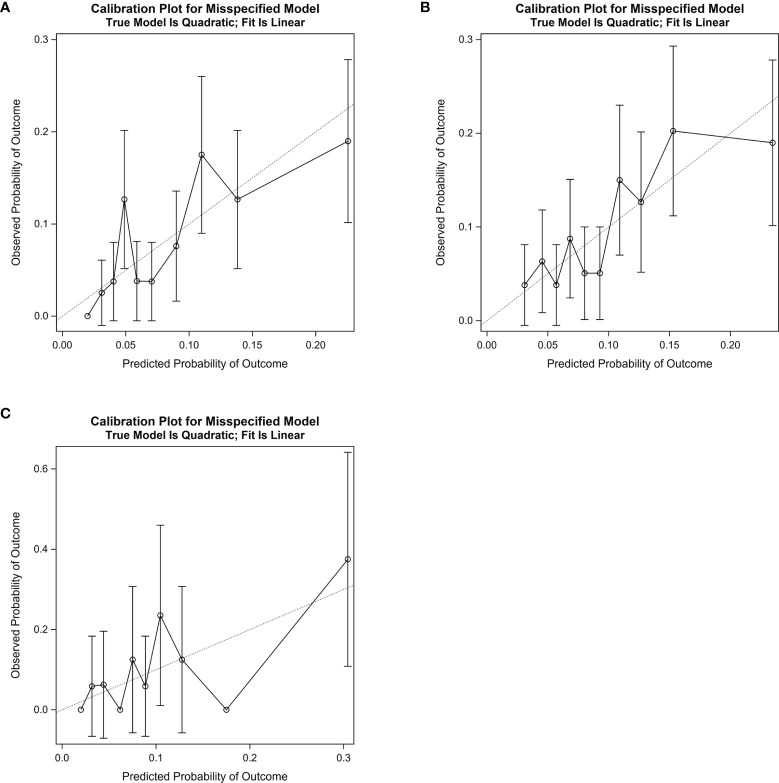
Calibration slope for the algorithm in the training group **(A)**, the internal validation group **(B)**, and the external validation dataset **(C)**. In the curve, the decile’s observed probability of developing postoperative pneumonia was plotted against corresponding predicted probability of developing postoperative pneumonia. The dotted line indicates the ideal calibration slope (Slope = 1.00). The calibration slope was 0.88 (95% CI: 0.37–1.39) in the training group, 0.90 (95% CI: 0.46–1.34) in the internal validation group, and 1.03 (95% CI: 0.24-1.83) in the external validation dataset.

Considering the calibration ability of the algorithm, the calibration slopes were 0.88 (95% CI: 0.37-1.39) in the training group (**Figure 5A**), 0.90 (95% CI: 0.46-1.34) in the internal validation group (**Figure 5B**), and 1.03 (95% CI: 0.24-1.83) in the external validation dataset (**Figure 5C** and **Table 4**). The *X*-intercept and *Y*-intercept of the algorithm were all close to 0 in the three datasets. The *R* ^2^ from the goodness-of-fit test showed the fitting of the line was good.

**Table 4:** Evaluation of the calibration ability of the algorithm.

Groups	Slope	95% CI	*X*-intercept	95% CI	*Y*-intercept	95% CI	*R* ^2^*
Training group	0.88	0.37–1.39	−0.011	−0.149–0.033	0.010	−0.042–0.062	0.67
Internal validation	0.90	0.46–1.34	−0.012	−0.126–0.032	0.010	−0.040–0.061	0.73
External validation	1.03	0.24–1.83	0.002	−0.361–0.070	−0.003	−0.107–0.102	0.53

*Goodness-of-fit test of the fitted lines.

CI, confident interval.

### Algorithm Classification

A predicted probability of developing postoperative pneumonia was capable of being calculated according to the algorithm in each patient. Then, three risk groups were created in the Thoracic Perioperative Database for Geriatrics according to the best cutoff values in the training group (cutoff = 8.00%), the internal validation group (cutoff=10.00%), and the external validation group (cutoff = 14.00%). The average cutoff value of the three groups was about 10.00%; thus, a predicted probability of less than 10.00% was classified into the low-risk group, a predicted probability of 10.00% to 20.00% belonged to the medium-risk group, and a predicted probability of more than 20.00% (twice the average prevalence) was the high-risk group (**Table 5**). The corresponding mean predicted probabilities were 5.65%, 13.54%, and 27.69% in the three risk groups and the corresponding mean actual probabilities were 5.14% (53/1031), 15.07% (71/471), and 25.30% (21/83), respectively (*p* < 0.001, Chi-square test). The predicted probabilities were similar to the actual probabilities among the three groups, which further confirmed the consistency and reproducibility of the algorithm.

**Table 5:** The classification of risk groups according to the algorithm among all patients in the thoracic perioperative database for geriatrics.

Groups	Patients (*n* = 1,585)	Probability of developing postoperative pneumonia		*p* ^a^
		Predicted	Actual	
Low-risk group (≧0.00% and <10.00%)	1031	5.65%	5.14% (53/1,031)	<0.001
Medium-risk group (≧10.00% and <20.00%)	471	13.54%	15.07% (71/471)	
High-risk group (≧20.00%)	83	27.69%	25.30% (21/83)	

a Indicates the p-value was from the Chi-square test.

### Investigation of Major Contributors

Principal components analysis was performed to explore major contributors to postoperative pneumonia and principal equations were given according to eigenvectors (**Supplementary File 2**). In the training group, seven principal components were developed and the cumulative contribution rate was 59.08% for the first three principal components, which indicated that the first three principal components were responsible for 59.08% information of all seven variables. According to the coefficients of the first three principal components, we might speculate that sex (coefficient = 0.62), intraoperative administration of colloids (coefficient = 0.67), and intraoperative administration of hormones (coefficient = 0.71) might be the major contributors to postoperative pneumonia since the three variables had the largest coefficient in the corresponding principal equations. Similar trends were observed in the validation group.

## Discussion

Our study developed an algorithm to predict the probability of developing postoperative pneumonia among elderly patients with lung cancer after video-assisted thoracoscopic surgery. In detail, the algorithm consisted of seven variables including sex, smoking, history of COPD, surgery duration, leukocyte count, intraoperative injection of colloid, and intraoperative injection of hormone. According to the discrimination and calibration abilities, this algorithm was a useful prediction model. Our study found that 9.15% (145/1,585) of patients suffered from postoperative pneumonia in the Thoracic Perioperative Database for Geriatrics, and 10.30% (17/165) of patients had postoperative pneumonia in the external validation dataset, and these numbers were consistent with other studies. Other studies reported that the prevalence of postoperative pneumonia was 3.6% (21) to 19.7% (15). This variation possibly could be explained by the population heterogeneity: the variety of age, different procedures, and diverse inclusive criteria. We also demonstrated that patients with postoperative pneumonia had significantly higher hospitalization expense as compared with those without postoperative pneumonia.

Several studies have pointed out that some variables were associated with postoperative pneumonia among lung cancer patients after surgery (14, 15, 22–33). More explicitly, in 2015, Simonsen et al. (22) concluded that advanced age, previous pneumonia, obesity, chronic pulmonary disease, alcoholism, atrial fibrillation, diabetes, male sex, and cardiovascular disease were risk contributors for postoperative pneumonia among patients treated with lung cancer surgery after analyzing a nationwide population-based cohort in Denmark. In 2011, Lee et al. (23) found that advanced age, intraoperative red blood cell transfusion, the presence of postoperative complications other than pneumonia, and low forced expiratory volume in 1 s/forced vital capacity were independent risk contributors for pneumonia after lung cancer surgery. In 2007, Shiono et al. (24) showed that advanced age, low forced expiratory volume in 1 s/forced vital capacity, and induction therapy were risk factors for pneumonia after lung cancer therapy in Japan. However, the above-mentioned studies enrolled lung cancer patients who were treated with various procedures and were also not especially for elderly patients. A small retrospective study demonstrated that the quality of surgery, namely, surgery time, was an essential contributor to postoperative complication among elderly patients after lung cancer lobectomy (25), but the procedure approach was also not clear and the sample size was 119 patients. Surgery type did play a role in affecting postoperative complications among lung cancer patients (21, 22). Theoretically, elderly patients were more vulnerable to postoperative complications due to low immunity and more comorbidity. Thus, in order to promote clinical significance and facilitate the development of the algorithm, our present study only enrolled the particular population. That was to say, we included elderly patients (age≧65 years) with lung cancer especially treated with video-assisted thoracoscopic surgery. To our knowledge, we developed the algorithm based on the largest population especially including elderly patients with lung cancer after video-assisted thoracoscopic surgery so far.

The present study showed that male sex, longer surgery duration, higher leukocyte count, larger intraoperative use of colloid, and intraoperative use of hormones were significantly associated with a higher rate of developing postoperative pneumonia especially among elder patients with lung cancer after video-assisted thoracoscopic surgery. Of the five significant variables identified by the LASSO method, two of them, i.e., male sex and longer surgery duration, were previously confirmed by other studies (22, 25, 26, 33). Since smoking status and history of COPD were previously confirmed to be significant with postoperative pneumonia (14, 22, 26, 27, 31), we also added the both to the algorithm. Yang et al. (15) demonstrated that intravenous infusion of excessive crystalloid within postoperative 24 hours could increase postoperative pneumonia. Our study pointed out that intraoperative administration of colloid had similar negative effects. It was suggested that intraoperative administration of hormone would be beneficial to prevent postoperative nausea and vomiting, but whether intraoperative administration of hormone could play a role in developing postoperative pneumonia remains controversial. In 2020, Newhook et al. (34) found that intraoperative administration of dexamethasone did not have any protective benefits regarding mitigating short-term infectious complications and long-term survival outcome among patients with pancreatic ductal adenocarcinoma, which was contradicted by the research conducted by Sandini et al. (35) and their colleagues in 2018. A Japanese researcher also held the view that perioperative use of methylprednisolone did not have protective effect for acute exacerbation of interstitial pneumonia among lung cancer surgery (36). Our study proved that intraoperative use of hormones (dexamethasone or methylprednisolone) might have a positive contributing role in developing postoperative pneumonia among lung cancer patients after surgery. We speculated that dexamethasone and methylprednisolone might fail to protect pulmonary surfactant morphology and even disturb the inflammatory response balance in the pulmonary because Mühlfeld et al. (37) once concluded that methylprednisolone failed to inhibit pulmonary tumor necrosis factor α (TNF-α), protect the pulmonary surfactant morphology, and maintain blood–air barrier integrity and lung function after cardiopulmonary bypass. However, two randomized clinical trials demonstrated that hydrocortisone therapy facilitated a decreased risk of hospital-acquired pneumonia among multiple trauma (38) and severe traumatic brain injury (39). The controversial conclusion between our present study and the two clinical trials could be explained by the variety of enrolled patients, timing of administration of drug, and pharmacological effects. Firstly, our study enrolled lung cancer patients treated with surgery, while the two clinical trials enrolled patients with trauma. Secondly, the hormone was administrated during surgery in our study, while it was intravenously infused during hospitalization in the two clinical trials. Lastly, dexamethasone and methylprednisolone had longer and stronger anti-inflammatory and anti-allergic effects as compared with hydrocortisone. Therefore, the effect of intraoperative use of hormones on preventing postoperative pneumonia still needs further investigation. A randomized, multicenter, double-blind, and superiority study addressing the problem of the perioperative administration of cortico-therapy on morbidity (including postoperative pneumonia) is also being conducted among non-cardiac major surgery but the results have not published yet (40).

Moreover, in the present study, the principal components analysis further demonstrated that sex, intraoperative administration of colloid, and intraoperative administration of hormone might be the essential contributors to postoperative pneumonia. Thus, some strategies to decrease intraoperative use of colloid and hormone might be considerably beneficial to prevent postoperative pneumonia. Besides, professional preoperative oral plaque control might also be a favorable measure to the prevention of postoperative pneumonia among lung cancer patients (41). In order to identify patients at a high risk of developing postoperative pneumonia and implement effective measures in advance, we classified patients into three groups according to the algorithm. Patients in the high-risk group had a mean actual risk probability of 25.30%, which was obviously higher than the mean actual risk probability of the whole population. Therefore, those patients need more and special attention to prevent postoperative pneumonia.

## Strength and Limitation

Our paper’s major contributions to the current literature included the following aspects. Firstly, to our knowledge, our algorithm was the first algorithm to especially predict the probability of developing pneumonia among elderly patients with lung cancer after thoracoscopic surgery. Besides, a large sample was included, external validation was performed, and a calculator was created in order to promote clinical utility. Secondly, the study found that intraoperative administration of colloid and hormone might be the essential contributors to postoperative pneumonia. Thus, the intraoperative administration of colloids and hormones need to be cautious to perform in the clinic, but it still needs large clinical trials to validate the conclusion in the future. Thirdly, we reviewed and summarized the recent literature reporting risk factors for predicting postoperative pneumonia among lung cancer patients (**Table 6**), which would increase the value of our work.

**Table 6:** A review about recent studies on risk factors for predicting postoperative pneumonia among lung cancer patients.

Years	Authors	Countries	Study date	Samples	Patient’s age	Diagnosis	Rates of thoracoscopic surgery (%)	Risk factors
2015	Simonsen et al. (22)	Denmark	1995–2011	7,479	Adult	Lung cancer	47.70%	Advanced age, previous pneumonia, obesity, chronic pulmonary disease, alcoholism, atrial fibrillation, diabetes, male sex, and cardiovascular disease
2011	Lee et al. (23)	Korea	2007–2009	417	Adult	Lung cancer	Approaches were not clear	Advanced age, intraoperative red blood cell transfusion, the presence of postoperative complications other than pneumonia, and low forced expiratory volume in 1 s/forced vital capacity
2007	Shiono et al. (24)	Japan	1992–2003	1,855	Adult	Lung cancer	Approaches were not clear	Advanced age, low forced expiratory volume in 1 s/forced vital capacity, and induction therapy
2013	Shiono et al. (25)	Japan	2000–2009	119	Elderly adult	Lung cancer	Approaches were not clear	Quality of surgery
2019	Yang et al. (15)	China	2016–2017	727	Adult	Malignant tumor (*n* = 634), benign tumor (*n* = 10), and others (*n* = 83)	100.00%	Intravenous infusion of excessive crystalloid within postoperative 24 h, body mass index ≥ 24.0 kg/m^2^, right lung lobe surgery
2018	Yendamuri et al. (26)	The United States	2008–2015	810	Adult	Lung cancer	91.50%	Mediastinoscopy, sex, history of COPD, smoker, and ASA class
2018	Agostini et al. (14)	UK	2012–2016	285	Adult	Lung cancer	100.00%	Current smoking^a^
2017	Liu et al. (27)	China	2014–2016	466	Adult	Lung cancer	98.30%	Older age, smoking and extent of excision of more than one lobe
2018	Dupont et al. (28)	France	2013–2015	200	Adult	Lung cancer	Approaches were not clear	Postoperative lymphopenia
2012	Díaz-Ravetllat et al. (29)	Spain	1999–2004	604	Adult	Lung cancer	Approaches were not clear	Body mass index, predicted postoperative FEV(1) <50%, and reintubation after surgery
2018	Shinohara et al. (30)	Japan	2007–2016	357	Adult	Lung cancer	41.12%	Age, oral steroid use, and lower-lobe resection
2021	Yao et al. (31)	China	2017–2018	726	Adult	Lung cancer	79.61%	Smoking, diffusing capacity for carbon monoxide, and the acute physiology and chronic health evaluation
2021	Motono et al. (32)	Japan	2002–2020	956	Adult	Non-small cell lung cancer	88.91%	Coexistence of asthma
2018	Kawaguchi et al. (33)	Japan	2004–2017	199	Elderly adult	Lung cancer	Approaches were not clear	Performance status, coronary artery disease, a history of cerebrovascular accident, restrictive ventilatory impairment, male sex, and interstitial pneumonia
2021	Song et al.	China	2012–2021	1,750	Elderly adult	Lung cancer	100.00%	Sex, smoking, history of COPD, surgery duration, leukocyte count, intraoperative injection of colloid, and intraoperative injection of hormones^b^

a The risk factor associating with postoperative pulmonary complication.

b the risk factors included in the algorithm.

ASA, American Society of Anesthesiologists; FEV, forced expiratory volume in one second; COPD, chronic obstructive pulmonary disease.

However, this study also had some limitations. Firstly, this study was retrospective in nature, so selection bias could not be entirely avoided. However, we enrolled patients *via* strict inclusion and exclusion criteria; thus, population homogeneity could be guaranteed to some extent, which was necessary to develop the algorithm. Secondly, some variables, such as postoperative lymphopenia (28), might have a role in developing postoperative pneumonia among elderly patients with lung cancer after surgery, but the variable was not analyzed in the present study because we aimed at predicting postoperative pneumonia using the variables collected during and before surgery, instead of using the postoperative variables. Furthermore, we used the LASSO logistic regression model to screen up to 30 variables including patients’ basic demographics, laboratory testing results, comorbidities, and preoperative medication, to name just a few. The variables analyzed in our study were relatively comprehensive and representative. Thirdly, histologic type was not analyzed in the study because, just as we mentioned above, the study tended to predict postoperative pneumonia with the variables collected during and before surgery. Histologic diagnosis was usually presented several days later after surgery. At that time, postoperative pneumonia might have already occurred. Besides, studies have confirmed that the histologic type might not influence postoperative pneumonia among lung cancer patients after surgery (23, 31). Therefore, this variable was not analyzed in the study.

## Conclusions

The algorithm can be a useful prognostic tool to predict the risk of developing postoperative pneumonia among elderly patients with lung cancer after video-assisted thoracoscopic surgery. Some interventions to tune surgery duration, leukocyte count, and intraoperative administration of colloid or hormone would be beneficial to prevent postoperative pneumonia.

## Data Availability Statement

The original contributions presented in the study are included in the article/**Supplementary Materials**. Further inquiries can be directed to the corresponding authors.

## Ethics Statement

The studies involving human participants were reviewed and approved by the Ethics Committee Board of the First Medical Center of Chinese PLA General Hospital (No. S2019-311-03). The ethics committee waived the requirement of written informed consent for participation.

## Author Contributions

All the authors conceived and designed this study together. YS, JL, YW, and BW performed data collection. YS, JL, ML, and BW performed data analysis, interpreted the results, and wrote the manuscript. YW, QF, and YG interpreted the results and revised the manuscript critically. and LT and WM supervised the research. All authors contributed to the article and approved the submitted version.

## Funding

This study was supported by the National Key Research and Development Plan (No. 2018YFC2001900 and No. 2018YFC2001901).

## Conflict of Interest

The authors declare that the research was conducted in the absence of any commercial or financial relationships that could be construed as a potential conflict of interest.

## Publisher’s Note

All claims expressed in this article are solely those of the authors and do not necessarily represent those of their affiliated organizations, or those of the publisher, the editors and the reviewers. Any product that may be evaluated in this article, or claim that may be made by its manufacturer, is not guaranteed or endorsed by the publisher.
