# Supplementary material for: An External-Validated Algorithm to Predict Postoperative Pneumonia Among Elderly Patients With Lung Cancer After Video-Assisted Thoracoscopic Surgery
Source: Front Oncol. 2021 Dec 14;11:777564. doi: 10.3389/fonc.2021.777564 (PMC8712479; doi:10.3389/fonc.2021.777564)
Supplement: Supplementary file 2 [file DataSheet_2.docx]

**Supplementary file 2**

In the training group, the first three principal components were given as follows:

Prin 1 = 0.62*sex + 0.60*smoking + 0.14*COPD + 0.30*surgery duration + 0.31*leukocyte count + 0.21*intraoperative administration of colloid - 0.08*intraoperative administration of hormone.

Prin 2 = -0.24*sex - 0.29*smoking - 0.04*COPD + 0.62*surgery duration - 0.04*leukocyte count + 0.67*intraoperative administration of colloid - 0.14*intraoperative administration of hormone.

Prin 3 = 0.04*sex - 0.05* smoking - 0.59* COPD + 0.05* surgery duration + 0.37* leukocyte count + 0.07* intraoperative administration of colloid + 0.71* intraoperative administration of hormone.

The eigenvalue and contribution proportion were given in **the following figure**.


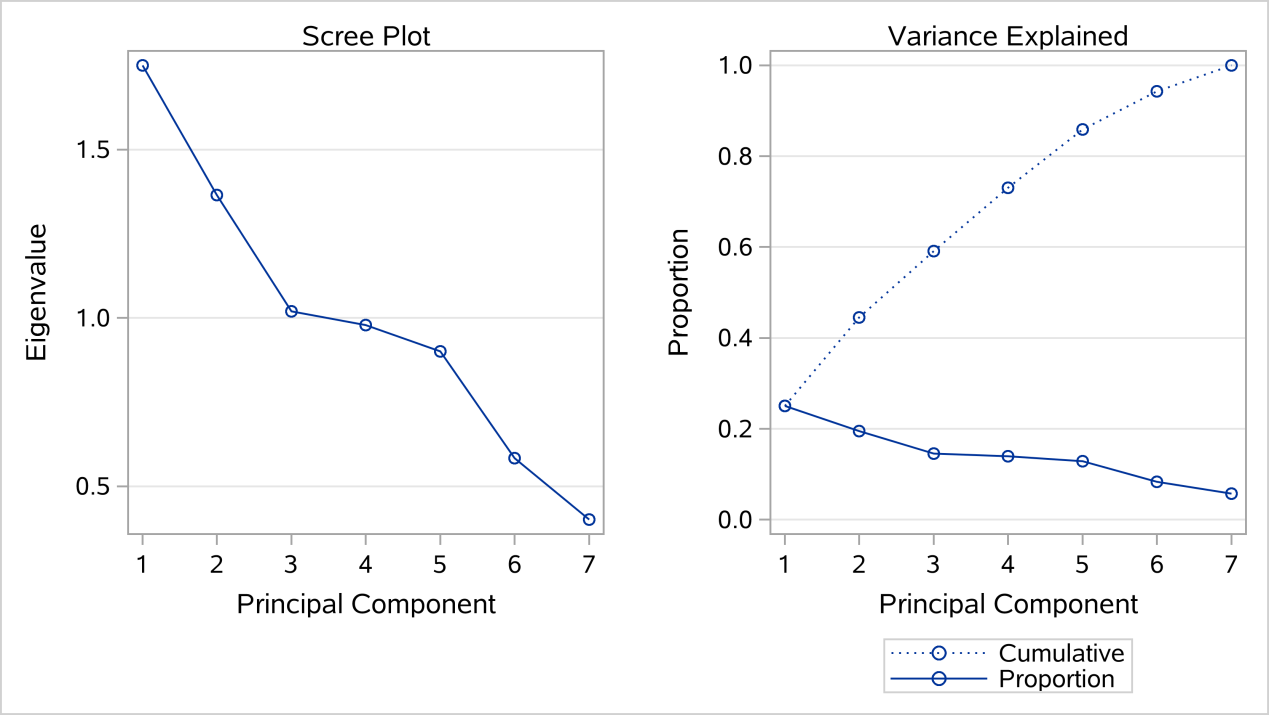


**Figure.** The left scree plot was plotted with eigenvalue against principal component. A higher eigenvalue indicates a higher contribution proportion. Prin 1 had the largest contribution proportion (25.00%), followed by Prin 2 (19.51%) and Prin 3 (14.57%), while Prin 7 had the smallest contribution proportion (5.73%). The right variance explained plot was plotted with proportion against principal component. The dotted line indicates the cumulative contribution proportion and the solid line indicates contribution proportion of each Prin.

In the validation group, the cumulative contribution proportion was 56.35% for the first three principal components, which indicated that the first three principal components were responsible for 56.35% information of the all seven variables. The first three principal components were given as follows:

Prin 1 = 0.63*sex + 0.62*smoking + 0.01*COPD + 0.30*surgery duration + 0.17*leukocyte count + 0.28*intraoperative administration of colloid + 0.15*intraoperative administration of hormone.

Prin 2 = -0.29*sex - 0.30*smoking + 0.07*COPD + 0.56*surgery duration - 0.13*leukocyte count + 0.61*intraoperative administration of colloid + 0.35*intraoperative administration of hormone.

Prin 3 = 0.05*sex + 0.04*smoking + 0.80*COPD - 0.28*surgery duration - 0.16*leukocyte count - 0.11*intraoperative administration of colloid + 0.49*intraoperative administration of hormone.

According to the above three principal equations, we might also speculate the similar conclusions in the validation group since sex (coefficient=0.63), intraoperative administration of colloids (coefficient=0.61), and intraoperative administration of hormones (coefficient=0.49) had the largest coefficient, which was similar to the results in the training group.

The eigenvalue and contribution proportion were given in **the following figure**.


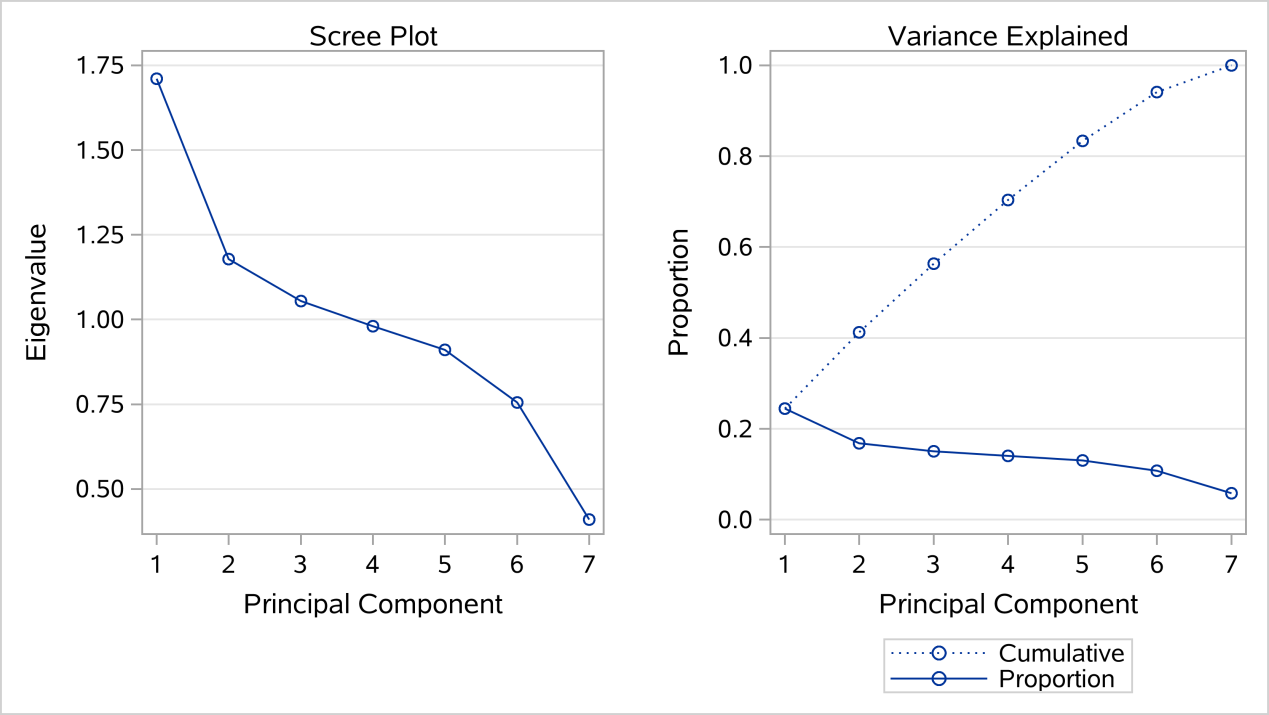


**Figure.** The left scree plot was plotted with eigenvalue against principal component. A higher eigenvalue indicates a higher contribution proportion. Prin 1 had the largest contribution proportion (24.44%), followed by Prin 2 (16.84%) and Prin 3 (15.07%), while Prin 7 had the smallest contribution proportion (5.85%). The right variance explained plot was plotted with proportion against principal component. The dotted line indicates the cumulative contribution proportion and the solid line indicates contribution proportion of each Prin.
